# Designing a mHealth clinical decision support system for Parkinson’s disease: a theoretically grounded user needs approach

**DOI:** 10.1186/s12911-020-1027-1

**Published:** 2020-02-19

**Authors:** L. Timotijevic, C. E. Hodgkins, A. Banks, P. Rusconi, B. Egan, M. Peacock, E. Seiss, M. M. L. Touray, H. Gage, C. Pellicano, G. Spalletta, F. Assogna, M. Giglio, A. Marcante, G. Gentile, I. Cikajlo, D. Gatsios, S. Konitsiotis, D. Fotiadis

**Affiliations:** 1 Faculty of Health and Medical Sciences, University of Surrey, Guildford, UK; 2 Department of Psychology, University of Bournemouth, Bournemouth, UK; 3 Department of Neurorehabilitation, Fondanzione Santa Lucia, Rome, Italy; 4 Fondanzione Ospedale San Camillo (I.R.C.C.S.), Parkinson’s Department Institute of Neurology, Venice, Italy; 5 University Rehabilitation Institute, Republic of Slovenia, Soča, Ljubljana, Slovenia; 6 Department of Material Sciences and Engineering, University of Ioannina, Ioannina, Greece; 7 Nurology, Faculty of Medicine, School of Health Sciences, University of Ioannina, Ioannina, Greece

**Keywords:** mHealth, M-health, Digital health, Clinical decision support system, CDSS, User needs, Parkinson’s, Mobile devices, Wearables

## Abstract

**Background:**

Despite the established evidence and theoretical advances explaining human judgments under uncertainty, developments of mobile health (mHealth) Clinical Decision Support Systems (CDSS) have not explicitly applied the psychology of decision making to the study of user needs. We report on a user needs approach to develop a prototype of a mHealth CDSS for Parkinson’s disease (PD), which is theoretically grounded in the psychological literature about expert decision making and judgement under uncertainty.

**Methods:**

A suite of user needs studies was conducted in 4 European countries (Greece, Italy, Slovenia, the UK) prior to the development of PD_Manager, a mHealth-based CDSS designed for Parkinson’s disease, using wireless technology. Study 1 undertook Hierarchical Task Analysis (HTA) including elicitation of user needs, cognitive demands and perceived risks/benefits (ethical considerations) associated with the proposed CDSS, through structured interviews of prescribing clinicians (*N* = 47). Study 2 carried out computational modelling of prescribing clinicians’ (*N* = 12) decision strategies based on social judgment theory. Study 3 was a vignette study of prescribing clinicians’ (*N* = 18) willingness to change treatment based on either self-reported symptoms data, devices-generated symptoms data or combinations of both.

**Results:**

Study 1 indicated that system development should move away from the traditional silos of ‘motor’ and ‘non-motor’ symptom evaluations and suggest that presenting data on symptoms according to goal-based domains would be the most beneficial approach, the most important being patients’ overall Quality of Life (QoL). The computational modelling in Study 2 extrapolated different factor combinations when making judgements about different questions. Study 3 indicated that the clinicians were equally likely to change the care plan based on information about the change in the patient’s condition from the patient’s self-report and the wearable devices.

**Conclusions:**

Based on our approach, we could formulate the following principles of mHealth design: 1) enabling shared decision making between the clinician, patient and the carer; 2) flexibility that accounts for diagnostic and treatment variation among clinicians; 3) monitoring of information integration from multiple sources. Our approach highlighted the central importance of the patient-clinician relationship in clinical decision making and the relevance of theoretical as opposed to algorithm (technology)-based modelling of human judgment.

## Summary points

- There has been limited evidence of the widespread implementation and acceptance of mHealth based Clinical Decision Support Systems (CDSS).
- The needs of the users of mHealth CDSS (i.e. clinicians) in terms of the cognitive demands of clinical decision making under uncertainty are not explicitly explored in the of design mHealth-based CDSS.
- Our research on clinicians’ user needs for the development of Parkinson’s mHealth-based CDSS is theoretically grounded in the psychological literature about expert decision making and judgement under uncertainty.
- It highlighted the central importance of the patient-clinician relationship in clinical decision-making and the relevance of theoretical as opposed to algorithm (technology)-based modelling of human judgment.

## Background

We are currently witnessing a paradigm shift in the global provision of healthcare, as new technologies are emerging to address growing challenges of cost, quality and continuity of healthcare provision. Rapid technological developments such as mobile health technologies (mHealth) and data analytics, offer solutions to the current unsustainable healthcare systems. “Mobile health” (mHealth), is defined as “medical and public health practice supported by mobile devices, such as mobile phones, patient monitoring devices, personal digital assistants and other wireless devices” [1]. mHealth is incorporated within the broader health informatics framework, though has emerged as a distinctive system in terms of context of use and acceptance. The pervasiveness, portability, convenience, immediacy and ubiquity of mobile devises enables not only continuous monitoring and communication required for the management of chronic conditions by healthcare providers at lower costs, but also patient empowerment to self-manage chronic conditions. Furthermore, the unique feature of mHealth is its ability to collect and connect medical, health-related and non-medical (e.g., behavioural) information that can feed into the existing health informatics systems such as electronic medical records and hospital information systems. This potentially innovative aspect of mHealth solutions, however, is currently largely confined to the non-healthcare context (e.g. lifestyle and public health applications) and lacking in acceptable frameworks for its wider adoption into the healthcare systems [2, 3] . Diffusion of new technologies necessitates clarifying the actual purpose of the innovations both from the point of view of healthcare system and the immediate needs of the users of the system, including patients and clinicians.

### The need for mHealth-based clinical decision-support system

For a health informatics system to be useful it needs to address all the complex cognitive needs of a clinician’s task. Medical decision making, especially concerning chronic conditions, is characterised by considerable complexity as it is often based on uncertain and incomplete information and therefore relies on the clinician’s expertise. The diversity of patient presentation and complexity of symptoms, time constraints, characteristics of patient–clinician encounter, and limitations of diagnostic tests all increase uncertainty in medical decision making, resulting in poor patient outcomes and increased costs [4–11]. Diagnostic uncertainty is ostensibly a subjective phenomenon based on the perceived lack of reliable information for treatment decisions [5].

The advances in mHealth such as data analytics and algorithm development based on continuous capture of a wide range of structured and unstructured patient-related data, can be harnessed to address such uncertainties through a Clinical Decision-Support System (CDSS) [12, 13], a software that can aid clinical decision making through careful matching of the patient individual factors (e.g., age, sex, ethnicity, computer literacy), their behaviour (in-situ and self-reported behaviour) and their current health status, with a computerised clinical knowledge base in order to provide patient-specific assessment and recommendations about clinical diagnosis/management [14]. A mHealth-based CDSS offers the potential to reduce medical errors [15] and improve the quality and efficiency of healthcare [16]. Nevertheless, actual application of such systems to date has been limited [17]. Several systematic reviews have examined the ability of mobile technology-based interventions designed to improve healthcare service delivery [18, 19]. Free et al. [18] identified a small improvement in outcomes relevant to clinical diagnosis, though the potential errors in reports and time taken to process data do caution against a wholesale and uncritical adoption of mHealth solutions. Bright et al. [20] similarly suggested that the evidence of their impact on clinical and economic effectiveness, user satisfaction and usefulness is ambiguous, with a range of studies variously demonstrating high, low and no difference in satisfaction and usefulness after introducing such CDSS’s in clinical decision making. The authors conclude that understanding the ways in which CDSS can meaningfully enhance specific clinical roles is a necessary next step towards the successful implementation of mHealth-based CDSS. In developing a mHealth CDSS, therefore, user-centred design is essential.

In software engineering, user-centred design comprises several key aspects: a) analysis of users’ characteristics and work environments; b) top level functional analysis of users’ goals, the domain structures needed for successful goal completion, and information flows within a system; c) tasks analysis; and d) representational analysis of user interface [21]. These software engineering-driven analytical strategies are mostly performed through ‘use cases’ - the industry standard for establishing system requirements. Whilst a use case approach facilitates requirement- gathering and subsequent description of the functional requirements of a system from a goal-based user perspective [22], it centres on the system architecture and system/service quality rather than the system user, is iterative and incremental, and mainly driven by the already established system prototype. By contrast, a fully user-centred approach *precedes* the development of the prototype and provides a nuanced understanding of intended users’ needs before the process of software development even commences.

In this paper, we report on research of user needs to inform and provide first principles for the development of the prototype of a mHealth CDSS [23] for a complex neurodegenerative condition – Parkinson’s disease (PD). Focusing on this particular chronic condition, our aim is to present a novel approach to establishing user needs, utilising a suite of methodologies that can be applied to a range of disorders. These methodologies are theoretically grounded in the psychological literature about expert decision making and judgement under uncertainty. Thus, they address the gap in the currently reported user studies for the development of CDSS which often lack a theoretical basis for their study design [24]. The unique characteristics of PD provide a fertile ground for the implementation of a CDSS based on mHealth, but due to its complexity, also present many challenges.

Parkinson’s was chosen because of its high prevalence in older adults and its complex and ever fluctuating symptom range. PD is the second most common neurodegenerative disorder in Europe with more than 1 million people living with the disease in Europe today and this number is forecast to double by 2030 [25]. The economic impact of the disease is substantial – the annual European cost is estimated at €13.9 billion [26]. Parkinson’s is a complicated disorder that most patients live with for many years and decades, becoming increasingly reliant on others to care for them in all aspects of life. The highly idiosyncratic presentation of the disease, its unpredictable progression, the plethora of possible symptoms both motor (tremor, bradykinesia, rigidity as well as gait/postural balance, speech disorders, swallowing disorders) and non-motor (cognitive disorders/dementia, depression and anxiety, Impulse Control Disorders, sleep disorders), as well as their daily and hourly fluctuation (e.g. motor on-off fluctuations, including freezing and dyskinesia’s, and non-motor fluctuations) render the disease particularly difficult to manage. The treatment is typically pharmacological, supplemented by occasional rehabilitative therapies, and in some cases, surgery. Clinical decisions are usually based on routine patient-clinician meetings (every 3–6 months). In that context, clinician reliance on patient and carers’ self-reports may be problematic. The presenting state of the patient may not indicate the extent and nature of functional impairments that are experienced at other points in time meaning that the clinician cannot gain a full and accurate picture of the patient’s status and the disease fluctuations that are crucial for appropriate titration of pharmacological treatment. Thus, a CDSS delivering a continuous stream of information via a mHealth solution has many obvious attractions, due to its pervasiveness, portability, context-sensitivity, immediacy and convenience [27].

### User-centred development of CDSS based on mHealth

A mHealth-based CDSS promises to deliver objective data about the patient’s healthcare status to the clinician in a timely manner [1] but, at the same time, risks increasing “technical uncertainty” [4], that is, the uncertainty due to the increased amount of available information, but not necessarily its utility, in making medical decisions. In addition, it has been shown that human beings tend to rely more on human judgement rather than on evidence-based algorithms, despite the latter being better predictors in many contexts (a phenomenon called “algorithm aversion” [28]).

An effective CDSS must be able to collect and link numerous types of patient-related data, be they physiological, pharmacological, behavioural and/or psychological measures. When developing the CDSS, a decision must be made about what types of data will be collected, connected and ultimately delivered to the clinician to optimise their judgment. Optimizing medication is the key for the management of PD and to support such functionality, the CDSS must employ algorithms that transform the stream of patient data into recognised symptoms (in order to recognise a patient’s state), and then to proposed decisions about the treatment plan. To develop accurate decision support systems, merely providing patient data is not enough; it needs to identify underlying expert models of decision making and compare the data against these expert models and clinical guidelines [29].

This includes an understanding of how clinicians interpret and respond to uncertain evidence. The latter is particularly relevant to PD given its multifaceted, complex and fluctuating nature [30–33]. It is important to understand whether and to what extent clinicians perceive a certain piece of evidence as informative and whether it is coming from a subjective information source (e.g., patient self-reports, diaries, questionnaires) or from an objective source e.g., wearable digital health technology, neuropsychological assessments, medical assessment techniques (such as Magnetic Resonance Imaging (MRI), blood samples) [34–36].

Early user (clinician) engagement with the design process and well-structured studies of user needs and their cognitive demands are therefore a necessary component of mHealth-based CDSS design and essential to increasing the usefulness, efficacy and acceptability of mHealth technologies. However, user engagement in the development of the CDSS is poorly reported and often given only a cursory role in the design. There is limited evidence of extensive exploration of clinicians’ decision-making processes, and how interventions will fit into routine clinical workflow. Previous design projects on CDSS for Parkinson’s, PERFORM [37], REMPARK [38] and SENSE-PARK [39], surveyed the factors influencing clinicians’ pharmacological decision-making – for instance, Serrano et al. [24] report on the participatory design process to achieve a consensus among patients, clinicians and technologists over the selection of a set of symptom domains to be continuously assessed. The reviewed projects, however, mainly focused on information needs and none examined in-depth the clinicians’ expert judgement models that are active in situ – the contexts characterised by the specific presentation of symptoms. Importantly, none of these approaches were grounded in the theoretical literature around task analysis, cognitive demands, expert judgments and cognitive processes under the conditions of the varying degrees of certainty. This limitation was addressed in the studies reported below.

### Aims and overall approach

**Fig. 1:**
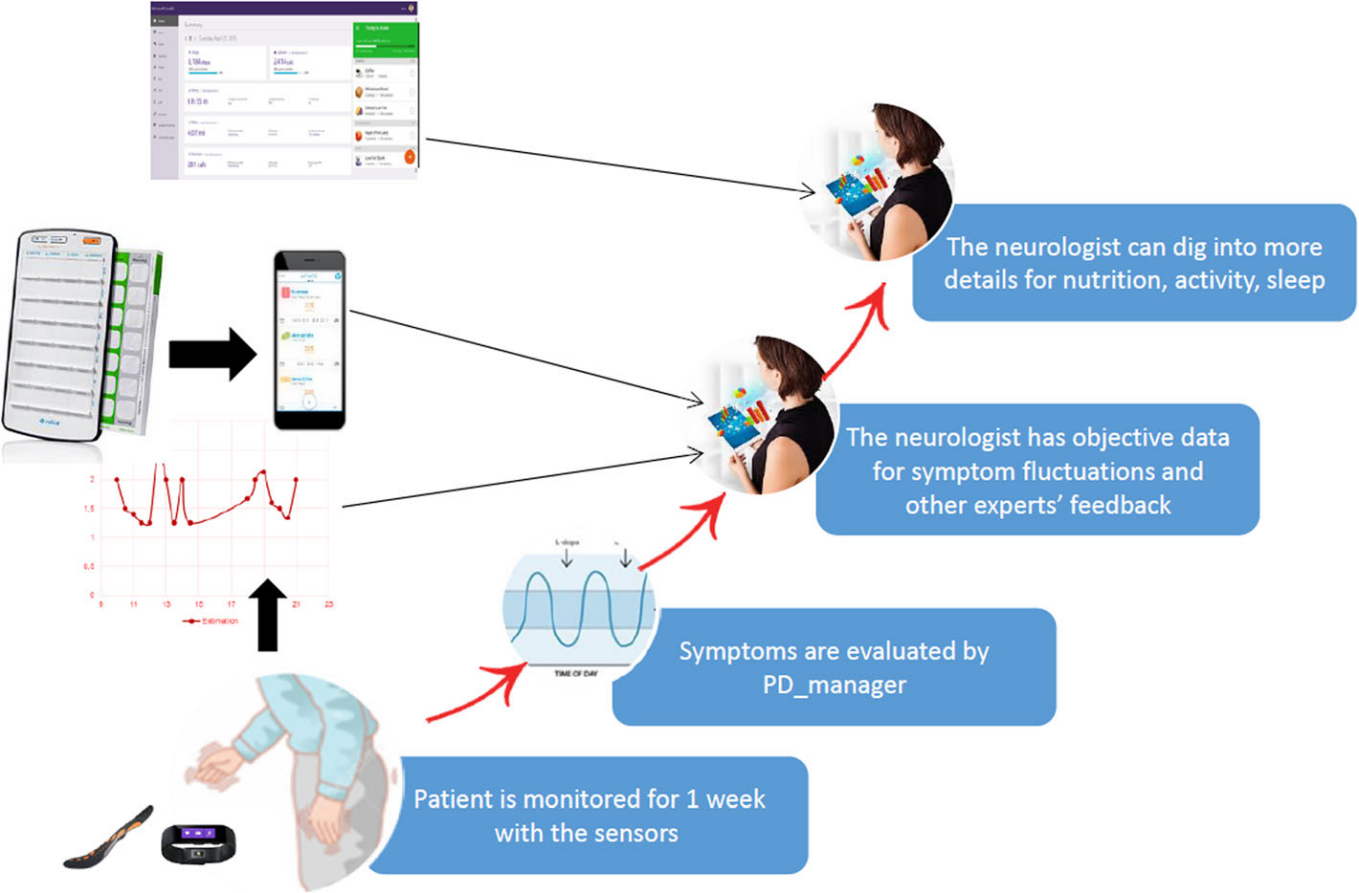
Implementation of the CDSS for the clinician

We report on PD_Manager, a case study of clinicians’ user needs for the development of a mHealth-based CDSS designed to help clinicians to monitor motor and non-motor symptoms using easily portable devices such as smart phones, wristbands and sensor insoles, worn by patients, to capture objective data about their fluctuating condition. The system is intended to combine machine learning and decision support methods with mobile and cloud-based approaches and share data via the cloud enabling clinicians to follow patients’ status closely. In this way, the PD_Manager CDSS enables continuous and accurate patient monitoring and PD symptoms assessment, delivered in a timely manner (see Fig. 1).

The aim of the mHealth-based CDSS is to enable the monitoring of patients, fluctuation of a range of symptoms or medication/treatment adherence, so that management plans can be evaluated and indications for modifications to medication regimens identified. The models for PD_Manager CDSS were developed through a combination of data mining of various PD symptoms; expert modelling using a qualitative multi-criteria method DEX, and assessment of models in terms of classification accuracy, transparency, correctness and completeness. This methodology is reported in a separate publication [40].

Due to the nature of the decision making (i.e. medication changes in a *progressive* chronic disease), which rarely if ever regards life threatening or emergency events, Quality of Services (QoS) aspects such as latency, priority and bandwidth were not critical for the development of the CDSS. Data loss was minimized as much as possible given that the system adopted commercial sensors. Other QoS aspects, such as power consumption were very important for the patient experience with the system and were taken into consideration both for the design of the system and for the pilot implementation. Data security was also addressed in the design and implementation of the mHealth system and was the main requirement for QoS within the PD_manager context.

In order to develop an initial set of principles for the CDSS, a series of user-needs studies were initially conducted, which we are reporting in this paper. The overall objective of our user-centred approach was to identify:

I. What types of decisions clinicians make when managing a patient with PD?
II. Information needs of the clinicians: What factors and their combination related to the patient symptoms inform their decisions?
III. Judgement under conditions of varying degree of certainty: What is the perceived value of self-reported patient data vs mHealth-generated data for a range of clinical decisions?

The following suite of three studies were conducted:

- Study 1 - Hierarchical Task Analysis (HTA) including elicitation of user needs, cognitive demands and perceived risks/benefits (ethical considerations) associated with the proposed CDSS.
- Study 2 - Computational modelling of clinicians’ decision strategies.
- Study 3 - Vignette-based study of the relative value of self-reported vs CDSS-generated evidence for clinicians’ decision making.

Study 1 utilised HTA through structured qualitative interviews, [41] as the framework for examining tasks associated with the clinical management of PD in terms of goals and sub-goals as well as the ethical considerations likely to arise when clinicians use mHealth-based CDSS. HTA, a core ergonomics methodology, is the established precursor for exploration of cognitive demands associated with human performance across a wide variety of domains and applications.

Study 2 is grounded in social judgement theory and related methodology [42]. This method has been used in many areas, and there has been extensive research on clinical judgement using it in a number of medical domains including studies of chronic heart failure [43], the management of rheumatoid arthritis [44], and decisions to use dialysis [45]. This approach aims to describe judgments through the combination of cues, such as signs or symptoms that are used to diagnose a cause or predict an outcome. Previous research on Parkinson’s disease has identified a comprehensive set of symptoms of relevance to managing the disease [24, 46]. Whilst this objective set of symptoms provides a useful framework of possibilities, which of these symptoms users actually use in their judgement process is a different question. A common finding across all domains of professional judgments, including medical judgments, is that the cues people actually use in their decisions are not the same as the cues they explicitly report using [47]. Experts use less information when making decisions than novices [48] and often disagree about their judgments [48]. Fully understanding user needs therefore requires an approach that captures the variation, context, and subjective weighting of each piece of information for individual users.

Study 3 builds on this recognition of the need to contextualise experts’ medical decision making by further exploring the situated nature of these judgements. It recognises the importance of understanding whether and to what extent clinicians perceive a certain piece of evidence as informative as a function of whether it comes from a subjective (e.g., patients’ self-reports, diaries) or from an objective (e.g., wearable technology, electrophysiological and neuroimaging techniques) source [35, 36]. In addition, we explore the uncertainty inherent in evaluating these types of evidence in situ by varying the degree to which the evidence from the two sources are congruent with each other. We deploy vignettes, a methodology that has already been successfully used to study how novices and experts make judgements in conditions of uncertainty based on patients’ self-reports as well as objective evidence, such as a physical symptom [49–51].

The data were collected between 2015 and 2017. Since the three studies collected data from clinicians (not patients), a formal ethical approval was not required within any of the national legislations (UK, Greece, Slovenia and Italy). However, within the UK, Research & Development approval from the hospital trusts - the two hospitals that allowed access to the clinicians - was obtained. For each study, written Informed Consent was obtained from the participants.

## Method

### Study 1: Hierarchical Task Analysis

Data were collected across four culturally diverse sites (UK, Italy, Slovenia and Greece) and from two core user groups: Prescribing clinicians (consultant neurologists, hospital doctors, general practitioners (GPs) and Parkinson’s disease nurse specialists (PDNS) and supporting clinicians (physiotherapists, occupational therapists, psychologists, speech therapists). A mixed-method approach was used whereby Prescribing Clinicians were interviewed one-to-one and Supporting Clinicians were either interviewed one-to-one or took part in a small focus group. The Prescribing and Supporting Clinicians recruited for the study are detailed by country (Table 1).

**Table 1:** Participant description

	UK *n* = 6	Italy *n* = 15	Greece *n* = 8	Slovenia *n* = 18	Total *N* = 47
Prescribing clinicians					
Consultant neurologist	2	4	8	7	21
Parkinson’s disease nurse specialist	3	–	–	–	3
General practitioner	1	5	–	3	9
Supporting clinicians					
Physiotherapist	–	2	–	2	4
Occupational therapist	–	1	–	2	3
Psychologist	–	3	–	2	5
Speech therapist	–	–	–	2	2

The added value of our cross-cultural approach is the ability to account for diverse models of clinical work organisation. Whilst working in multi-disciplinary teams for complex neurological diseases such as PD is standard practice in state medical systems such as UK, Italy and Slovenia, the Greek system is characterised by a predominant role of consultant neurologists within the state system, supplemented by specialist treatment in the private system.

The first step was to clearly define the overall task under analysis and the specific purpose for that analysis. Within this study the top-level task and goal were collectively defined as:

***‘****Effective management of Parkinson’s disease in order to identify where the proposed outputs of the PD_manager project can add value to the task’.*

There followed a desk-based review of current practice from the literature resulting in the development of a preliminary Hierarchical Task Diagram (HTD). Utilising this preliminary HTD as a stimulus, data were collected from the prescribing and supporting clinicians via structured qualitative interviews (locally, within each of the 4 countries) to expand our understanding of the task, to determine task sub-goals and ultimately to achieve full task decomposition. During the interviews clinicians were asked to reflect on the preliminary HTD and suggest any changes/amendments to accurately reflect the broadest range of tasks they typically undertake. Cognitive demands, decision-making strategies, perceived usefulness of symptomatic data, perceived importance of patient adherence, supporting therapies and beliefs about mHealth-based CDSS (including ethical considerations) were elicited in parallel by a generalist approach within the interview to allow us to encompass the whole domain more effectively.

### Study 2: Modelling clinicians’ decision strategies

The aim of this study was to develop a model of how clinicians use information from patients with Parkinson’s disease in the management of PD using a factorial survey design. We elicited the decision-making strategies of clinicians, describing how strongly weighted each of the factors are within their overall judgements. Prescribing clinicians assessed 24 cases, each one describing the symptoms of a different patient. For each case, clinicians were asked to make judgements about the patient. Specifically, they made judgements about changing the care plan, change to Levodopa, and change to dopamine agonist. In each case, 13 key symptoms or factors in the assessment of Parkinson’s disease were varied systematically. These were: bradykinesia, rigidity (stiffness), tremor, gait and balance, sleep, cognitive functioning, depression, constipation, motor fluctuations, dyskinesia, impulsivity, age and employment status. The changes in these symptoms were described to clinicians in terms of a comparison to a consultation three to 4 months previously. That is, each of the symptoms was described as ‘better’, ‘same’, or ‘worse’ than three to 4 months previously. Two contextual factors were: age (either ‘55 years old’ or ‘75 years old’) and employment status (either ‘employed’ or ‘retired’). Participants were informed that in all cases they should assume that the patient with Parkinson’s disease was currently taking Levodopa and a dopamine agonist, the disease duration is 5–10 years and their disease severity is stage 3 on the Hoehn and Yahr scale [52] (please see the Table 2 for an example of a case).

**Table 2:** An example of the Study 2 case

Demographics	Motor Symptoms		Non-motor symptoms		Treatment adverse effects	
75 years old, retired	Bradykinesia	Worse	Sleep	Better	Motor fluctuations	Same
	Rigidity (stiffness)	Worse	Cognitive functioning	Same	Dyskinesia	Better
	Tremor	Worse	Depression	Same	Impulsivity	Better
	Gait & balance	Same	Constipation	Better		

### Study 3: vignette study of value of different types of information for clinicians’ diagnostic strategies

The study aim was to investigate clinicians’ decision making about treatment and care plans based on the relative utility of subjective (reported by a patient with Parkinson’s disease) or objective (digital health) information. Prescribing clinicians completed an online questionnaire with 15 vignettes describing situations involving hypothetical patients with Parkinson’s disease (the surnames used in the vignettes were not of real patients). For all vignettes, the main information about the patients was kept constant: they were described as older (between 66 and 70 years old), living with their partner, with their symptom severity at Hoehn and Yahr stage 3, disease onset was 7 years ago, and the current therapy as prescribed for the past 6 months. Furthermore, we randomly varied the patients’ gender across the 15 vignettes.

Two within-participant variables were manipulated in these vignettes: information type (5 levels) and symptoms/signs (3 levels). We manipulated the type of information presented to the clinicians as follows (see Table 3): subjective information (patients’ self-report); objective information (digital health data); congruent subjective and objective information (both sources suggested a decline in patients’ symptoms/signs); incongruent subjective and objective information (one indicating a decline and the other indicating an improvement).

**Table 3:** Information about the manipulated information type variable in Study 3

Variable	Levels				
Information type	Subjective (self-report)	Objective (device outcome)	Subjective & objective –congruent	Subjective & objective –incongruent Self-report: *decline* Devices: *improvement*	Subjective & objective –incongruent Self-report: *improvement* Devices: *decline*

For each information type, we devised three different scenarios where the main difference was the symptom/sign: (1) bradykinesia [53, 54], and impaired dexterity, (2) gait and postural stability, and (3) sleep. The following is a sample vignette presented to participants depicting a case of incongruence between the patient’s self-report (indicating a decline in gait and postural stability) and a sensor insole (indicating an improvement):

*“Mr Briggs is 69 years old, retired, and he lives with his wife. He suffered from the first symptoms of PD approximately seven years ago (Hoehn and Yahr stage 3).*

*In a consultation with you, the patient tells you that he feels that his condition has* worsened *and he has been experiencing more falls and gait difficulties.*

*Data from the patient’s sensor insole, a device which has captured information on his PD symptoms, show that he has* improved *and has experienced a decreased number of falls and gait difficulties since his last clinical consultation.*

*The patient has been receiving medication and physiotherapy treatment over the last 6 months.”*

After reviewing each vignette, participants were asked to select a decision about the therapy (“Based on this information, how likely would you change the patient’s care plan?”) using a 7-point Likert scale (1 = very unlikely, 7 = very likely), and about the confidence in the decision (“How confident are you in the answer you have just given?”, 1 = not at all confident, 7 = very confident). Finally, they were asked to indicate what type of data they would find more useful in order to increase confidence in their decision – subjective (PD patient’s self-report) or objective (devices-generated) data (“Given your decision, how useful would you find it to seek for more information from the patient or the devices to increase your confidence?”). They used two 7-point Likert scales (1 = not at all useful, 7 = very useful) to provide their answer for both the self-report and the device-generated data.

After completing the vignettes, participants’ socio-demographic data (including age, gender, and nationality) were collected. The survey lasted approximately 30 min.

## Results

### Study 1: Hierarchical Task Analysis

**Fig. 2:**
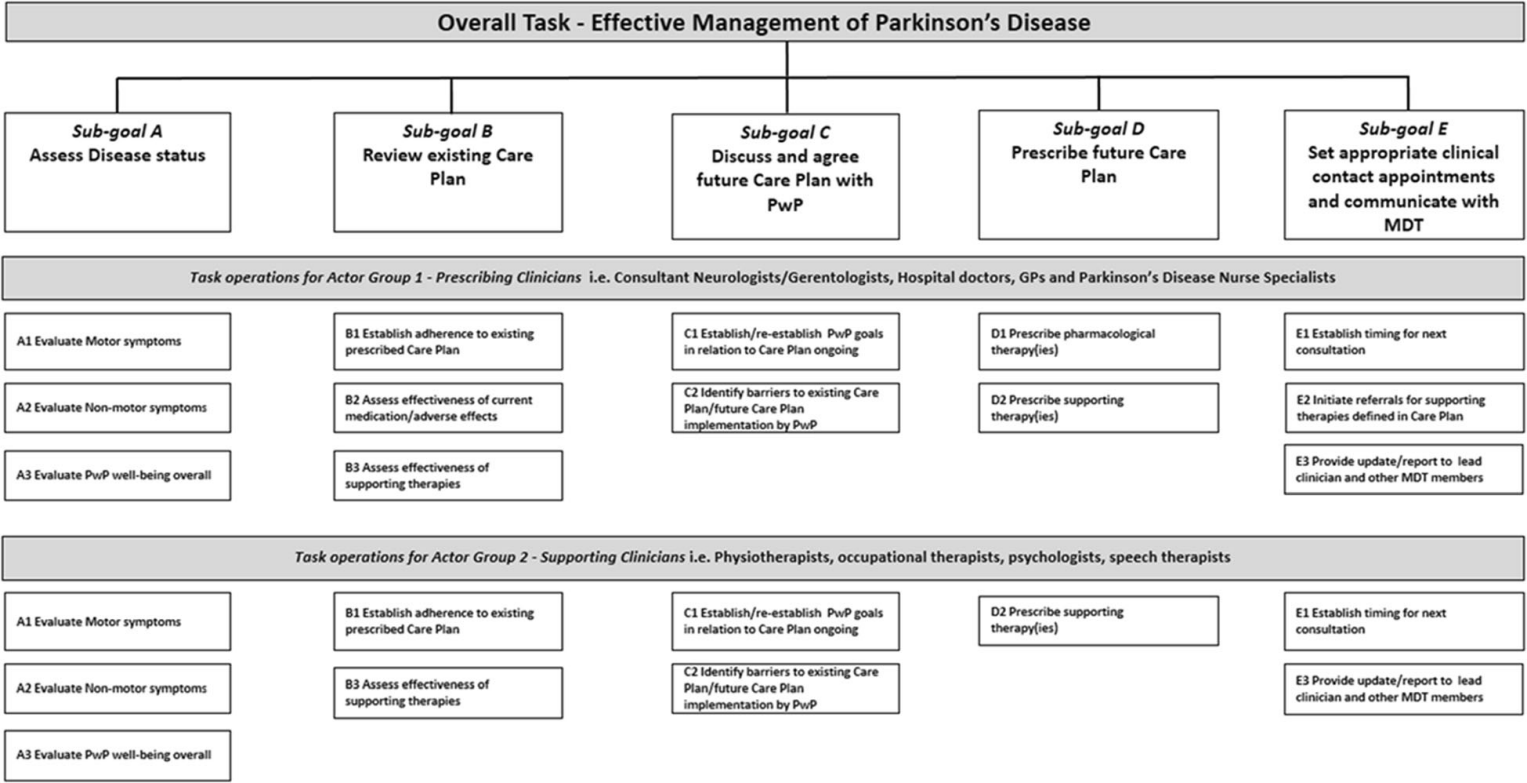
Hierarchical Task Analysis Diagram reflecting sub-goals and task operations for both Prescribing and Supporting Clinicians

Analysis of the qualitative data comprised a mixture of summative content and thematic analysis [55]. Coding structures were developed to guide the analysis of the data and ensure a harmonized and standardized approach to analysis of the data across the different countries. The resultant data were used to develop an updated version of the Hierarchical Task Diagram validated by the interview process (Fig. 2).

The majority of clinicians interviewed in this study operated in an environment with little or no access to continuous objective data on PD motor symptoms to support their clinical decision making, even at its most basic level. Therefore, the provision of this type of data in graphical form by the mHealth-based CDSS was deemed to be useful. Having identified the cognitive demands and difficulties clinicians experienced when managing patients and their expressed needs (Appendix 1) as well as the perceived usefulness of data on the various motor and non-motor symptoms (Appendix 2), a set of user-derived requirements for the mHealth-based CDSS design were proposed (Appendices 3 and 4).

Approaching the development of user needs using this methodology, the outcomes of this study suggested a move away from the traditional silos of ‘motor’ and ‘non-motor’ symptom evaluations and suggest that presenting data on symptoms according to goal-based domains would be the most beneficial approach. This innovative goal-based domain approach including: Diagnosis indicators; Disease progression indicators; Pharmacological decision support; Patient safety; Overall QoL indicators; Physiotherapy/Occupational therapy; Psychology and Speech therapy (Appendix 3) would facilitate the collation/integration of the relevant non-motor symptom data with the motor symptom data for each of these domains in more targeted and user-friendly interfaces. Furthermore, the data suggested that the most useful symptoms to evaluate should be driven by the importance the patient themselves attribute in terms of their overall QoL. Therefore, it was suggested that design should deliver functionality to allow for the creation of personalised user interfaces that provide flexibility for clinicians/patients to identify and select which symptoms they wished to consider at any one time. The availability of such a flexible system should allow clinicians to focus on the data they needed to engage with, to better facilitate patient engagement in shared decision making and potentially achieve better adherence and clinical outcomes.

Finally, the content analysis of attitudes towards the technology and ethical issues indicated that the participants were not aware of any ethical issues associated with the mHealth-based CDSS. There seemed to be a general agreement that the systems needed to manage privacy concerns were already in place such as, for instance, data management processes and consent forms. However, when asked whether they were aware of any governance guidelines /codes of practice regarding the collection and transmission of patient data using technology, none of the participants were able to refer to these systems within the context of their work.

### Study 2: Modelling clinicians’ decision strategies

Twelve prescribing clinicians (7 males and 5 females), with a mean age of 45.58 years, (*SD* = 8.76) from 4 countries: Italy (*n* = 3), Greece (*n* = 2), Slovenia (*n* = 3) and the UK (*n* = 4) took part in the study. They were consultant neurologists (*n* = 9), consultant gerontologist (*n* = 1) and general practitioners (n = 2). These clinicians saw a mean of 172.67 patients with Parkinson’s disease per year (*SD* = 215.26). Multiple regression was used for each clinician to determine the weighting of each of the factors on each of the judgements. The judgement patterns were calculated for each participant individually based on their responses to the judgement task. Each of the factors within the cases was coded as an independent variable and the judgements made about each case were the dependent variables. The *R*^*2*^ value for the regression model describes how well the factors explain the judgements made and the standardised beta weights for each factor describe the weighting of that factor in the judgement. Table 4 presents the beta weights for each factor in each judgement and the *R*^*2*^ for each model.

**Table 4:** Standardised beta weights for the mean response to identify the factors predicting changes to treatment

Factor	Care Plan	Levodopa	Dopamine Agonist
Bradykinesia	.36	.31	.14
Tremor	.51	.47	.44
Gait	−.02	.16	−.04
Dyskinesia	.19	−.29	.31
Motor Fluctuations	.44	.44	−.16
Sleep	.10	.04	.19
Cognition	.23	.27	.00
Impulsivity	−.09	−.07	−.36
Depression	.22	.08	.27
Constipation	−.02	−.10	.06
Rigidity	.35	.41	.34
Age	−.31	−.07	.00
Employment	−.37	−.29	.26
*Model R*^*2*^	*0.82*	*0.90*	*0.70*

N.B. for symptoms, a positive beta weight implies an increased likelihood of change to the care plan, or an increased level of medication, in response to worsening symptoms

Across the different models, findings showed that clinicians were more likely to change the care plan and increase the level of medication when presented with worsening symptoms, with the exception of a reduction in Levodopa with worsening dyskinesia and a reduction in dopamine agonist with worsening impulsivity. Analysing the findings in more detail provides some more specific results. Across the different judgements, the factors that dominate the decision are motor symptoms: bradykinesia; rigidity; motor fluctuations; and tremor. Non-motor symptoms such as depression, impulsivity and cognitive function explained some variance in the judgements, albeit less than bradykinesia, rigidity, motor fluctuations and tremor, but sleep and constipation had very little effect on the judgements made. Some factors were weighted more similarly by all clinicians, e.g. tremor, whereas others were weighted more differently, e.g. bradykinesia. Furthermore, the study shows that different factor combinations (e.g. symptom, age, employment status) are important when making judgments about different questions (e.g. changing the care plan, change to Levodopa, and change to dopamine agonist).

### Study 3: Vignette study of value of different types of information for clinicians’ diagnostic strategies

Participants were *N* = 18 (10 females; 8 males) prescribing clinicians practicing in Greece (8), Italy (5), the UK (4), and Slovenia (1). Their mean age was 41.94 (*SD*_age_ = 7). They were consultant neurologists (13), general practitioners (2), consultant gerontologist (1), neurology resident (1), and researcher (1).

**Fig. 3:**
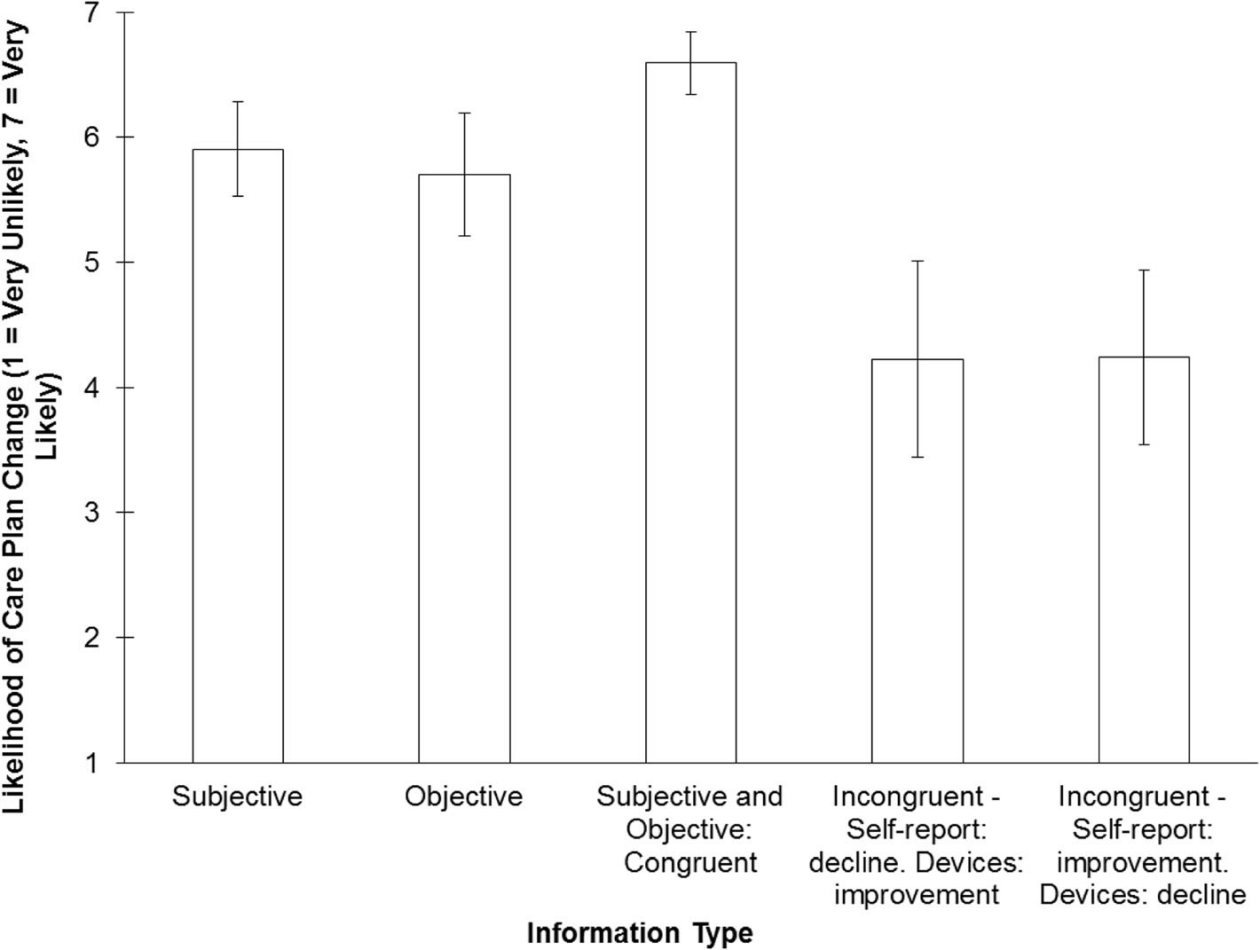
Mean likelihood of changing the patients with Parkinson’s Disease care plan as a function of the different pieces of information presented to participants in the scenarios (i.e., self-report only, devices’ outcome only, consistent (congruent) or conflicting (incongruent) information from both sources). Error bars represent 95% confidence intervals

In our analyses, given that our main variable of interest was Information Type, we averaged participants’ responses across the three primary symptoms. The results indicated that the clinicians were equally likely to change the care plan based on information from the patients’ self-report and the devices about the decline of their condition. In addition, they were more likely to revise the care plan when they received congruent information from both sources and less likely to revise the care plan when they received conflicting information from both sources (Fig. 3).

The findings about clinicians’ confidence in their decision mirrored these findings. Clinicians were equally confident in changing the care plan when receiving information from only one information source, most confident when the information from both sources was congruent and least confident when it was incongruent.

## Discussion

As highlighted by Castro et al. [56] a crucial question to ask in the development of a CDSS is: What is the system intended for? Therefore, the early understanding of user needs is the most important aspect of system development. And yet, the predominance of the software engineering-driven CDSS development with a main focus upon *information needs* of a user and *use cases* based on the already established prototypes [24], is unlikely to provide a full account of the social (e.g., the interactions between patients, caregivers, and clinicians; time constraints) and psychological (e.g., clinicians’ judgement and decision-making strategies) realities within which the technology will be operationalised. We addressed these shortcomings in our research.

Our approach to user studies was developed to identify the core principles of design to guide an early development of the CDSS. Based on our findings, we can formulate three main principles of design with some important theoretical and practical implications: 1) enabling shared decision making between the clinician, patient and the carer; 2) flexibility that accounts for diagnostic and treatment variation among clinicians; 3) monitoring of information integration from multiple sources.

### Implications for the system design

Our study highlighted that the primary goal of the system should be to enable shared decision making between the clinician (e.g., neurologist, PD nurse, physiotherapist), patient and the carer [57, 58]. This is evident in several findings: our HTA interview study (Study 1) has highlighted that the needs of clinicians should be conceptualised in terms of how the system is likely to support the clinician-patient interaction. The practical implication of this finding is that the design should be driven by the patient-specific goal-based domains rather than the expert-defined symptom categorisation. Study 1 identified that the most useful symptoms to evaluate should be decided by the importance patients themselves attribute to those symptoms in terms of their overall QoL. A mHealth-based CDSS should therefore be conceptualised as a vehicle that facilitates co-production of disease management solutions and should be embedded within the context of patient-clinician interaction.

The shared nature of decision making extends the approach to decision making applied in Study 2. Previous work applying social judgement theory to clinicians’ decision making uses a paradigm in which an individual clinician is presented objective information and forms a judgement about the patient. From this analysis their personal evaluation can be inferred [43–45]. The context of this study demonstrates that an individual clinician’s weighting of cues is not the only consideration. Instead, the weighting of symptoms and desirability of outcomes may emerge through the interactions of clinicians, patient, and carer. Study 3 extends the approach to decision making, demonstrating how the weighting of this information is also influenced by its source – the patient’s subjective report or digital device, thus highlighting again the key role of the clinician-patient interaction and communication. The unique context of mHealth devices, in comparison to digital devices in more established settings such as hospitals, further influences their interpretation.

Secondly, our suite of studies highlighted that the system should take into account diagnostic variation [5] as well as treatment variation among clinicians. In other words, a CDSS should allow different judgement patterns for the disease management to operate for similarly presented symptoms. The evidence for this principle emerged from Study 2, which identified similarities in the way clinicians evaluate symptoms, which, nevertheless, may be linked to significant variations in the way in which the disease is ultimately managed. Whilst the study did not report on the actual causal models underpinning this variability, it indicated that different users are likely to prefer different information in management of the disease. This variation is potentially driven by not only symptoms presentation but also the immediate as well as broader context of patients’ lives. Again, this illustrates that it is not possible to define a set of the most important factors for all cases, but rather, that the system must have an inbuilt flexibility to allow the operation of different solutions representing co-production by clinician and patient, which would improve the completeness and usefulness of the model. This flexibility should be combined with a method to optimize the quality and overall performance of the decision-making process. The recent developments in quality of service-based web- service selection [59] may be one way forward in the identification of the most important factors that address user needs and increase user satisfaction whilst maintaining system flexibility.

Finally, our work pointed to the necessity for a system to fully account for and continuously monitor the shortcomings, risks and compromises inherent in integrating quantitatively different types of information from multiple sources. Study 3 provides evidence that the combination of information from the patient and from the mHealth devices can differentially influence experts’ decisions and their confidence in those decisions. This is demonstrated by the statistically significant differences between the conditions in which both types of information were presented (i.e., the congruent and incongruent conditions) and the conditions in which only one source of information (either the patients’ self-report or the devices’ outcomes) was available. The lack of difference, both in terms of their impact on the care plan revision and the confidence levels, between the patients’ self-reports and devices’ outcomes suggests that clinicians consider the - ostensibly subjective - information provided by patients as equally useful and trustworthy as the objective information generated by the devices. This finding about the clinicians’ reliance on subjective as well as objective evidence is inconsistent with the view that technology-based evidence is unbiased, more ecologically valid, and more reliable than subjective assessment [32, 33, 60, 61]. It supports our first principle, however, that puts an emphasis on the patient-clinician relationship as a focal point of the CDSS.

This finding bears on the actual implementation of a mHealth-based CDSS. In particular, it would be important to identify conditions, risks and mechanisms that could give rise to conflicting information and the impact that this could have on disease management and patients’ care. Mechanisms and procedures could then be put in place to deal with these situations as well as to prevent circumstances in which clinicians are reluctant to change the treatment (as it was the case in the presence of conflicting information from the patients and the devices in Study 3). These are situations that can generate technical uncertainty, whereby paradoxically the knowledge is inadequate, even if data and technology are available [4]. Furthermore, uncertainty could be used to justify risk discounting or inaction [62, 63], mHealth based CDSS should therefore not only provide appropriate and reliable evidence to aid clinical judgments, but also aim to reduce the uncertainty inherent in natural decision-making contexts experienced by clinicians –when a clinician and a patient are actually interacting.

Finally, we would like to comment on one surprising finding indicating that, at the time of the interviews, the clinicians appeared to have no ethical concerns associated with the advent of machine learning and mHealth into their clinical practice. Ethical judgments of clinicians are currently covered by the established principles of medical decision making - the Hippocratic Oath of “do no harm” and the Helsinki declaration [64] of having appropriate oversight of data management and the need to seek patient consent. However, the huge advances in the field of Artificial Intelligence and mHealth must be accompanied by rapid education to prepare clinicians for assessing the ethical implications of using these systems in their interactions with patients. Of particular concern is the issue of the accountability of technology – both in terms of its ability to provide trusted and relevant information, but also in terms of its ability to recount and feedback the way in which algorithms are providing the data for the mHealth-based CDSS (explainability). This directly links to the issue of how the assessments of accountability and responsibility of the clinician can be established in situations where they may not be able to assess the reliability or usefulness of information that technology provides, and when they may not be able to meaningfully assess the consequences of their judgments. It is the latter issue that calls for clinicians’ critical awareness of the nature of such technology and the possible biases that may be perpetuated throughout the life cycle of a technological system. Engaging clinicians in these discussions is essential in order to prevent the deterioration of patient trust and ensure effective healthcare delivery based on any future mHealth CDSS.

Future research should seek to explore the relevance of the core principles for mHealth-based CDSS development identified by this study in the Parkinson’s domain in other chronic disease domains. We focused on a specific, multifaceted, and fluctuating neurodegenerative disease, but it is possible that for each chronic-disease domain these core principles may need to be tailored to some degree. However, the utilisation of the theoretical and methodological framework described here would facilitate the identification of any domain-specific issues.

## Conclusions

Being guided by psychological theory enabled us to move beyond mere information -gathering to develop core principles for designing a Parkinson’s mHealth based CDSS. We developed a novel approach to address the questions of the system’s core purposes, which provided a more nuanced accounting of clinician judgment in situ and helped formulate a vision for the mHealth-based CDSS system functionalities. This vision highlights the central importance of the patient-clinician relationship in clinical decision-making and the relevance of theoretical as opposed to algorithm (technology)-based modelling of human judgment. It challenges the assumption that augmented decision making - based on the operation of algorithms - is the best solution to better clinical decision making, instead establishing human decision making as central in the increasingly machine- and data-driven world of healthcare.

## Data Availability

The datasets generated and/or analysed during the current study are not publicly available. This is because the written consent from the participants to re-purpose the anonymised data was given under the condition that the data would only be made available if relevant legal, professional and ethical approvals were provided. Anonymised data is available from the corresponding author on reasonable request.

## Appendix 1

**Table 5:** Cognitive Demands Table

Task Operation	Cognitive element	Difficulties experienced by clinicians and their relevance to each actor group	
		Prescribing clinician	Supporting clinician
A1 Evaluate Motor Symptoms.	Establishing the extent to which the PwP’s motor symptoms have improved/ declined/ remained stable since the last clinical consultation.	A1.1 The reliability of the PwP/Caregiver’s self-report can be dependent on the social, educational, or cognitive status of the PwP/Caregiver. A1.2 Lack of time in consultation with PwP to elicit information on all symptoms. A1.3 Important changes in symptoms can be masked by the PwP/Caregivers tendency to focus on the one symptom that is most salient/problematic for them at that moment. A1.4 Clinical examination/observation of PwP’s motor symptoms during the consultation may not reflect the symptoms they are experiencing at home/in their daily life. A1.5 Dyskinesia and Tremor are sometimes confused by PwPs/Caregivers in self-reports.	A1.1 The reliability of the PwP/Caregiver’s self-report can be dependent on the social, educational, or cognitive status of the PwP/Caregiver. A1.2 Lack of time in consultation with PwP to elicit information on all symptoms. A1.3 Important changes in symptoms can be masked by the PwP/Caregivers tendency to focus on the one symptom that is most salient/problematic for them at that moment. A1.4 Clinical examination/observation of PwP’s motor symptoms during the consultation may not reflect the symptoms they are experiencing at home/in their daily life.
A2 Evaluate Non-Motor Symptoms.	Establishing the extent to which PwP’s non-motor symptoms have improved/ declined/ remained stable since the last clinical consultation.	A2.1 The reliability of the PwP/Caregiver’s self-report can be dependent on the social, educational, or cognitive status of the PwP/Caregiver. A2.2 Lack of time in consultation with PwP to elicit information on all symptoms. A2.3 Important changes in symptoms can be masked by the PwP/Caregivers tendency to focus on the one symptom that is most salient/problematic for them at that moment. A2.4 Clinical examination/observation of PwP’s motor symptoms during the consultation may not reflect the symptoms they are experiencing at home/in their daily life.	A2.1 The reliability of the PwP/Caregiver’s self-report can be dependent on the social, educational, or cognitive status of the PwP/Caregiver. A2.2 Lack of time in consultation with PwP to elicit information on all symptoms. A2.3 Important changes in symptoms can be masked by the PwP/Caregivers tendency to focus on the one symptom that is most salient/problematic for them at that moment. A2.4 Clinical examination/observation of PwP’s motor symptoms during the consultation may not reflect the symptoms they are experiencing at home/in their daily life.
A3 Evaluate PwP well-being overall.	Identifying which symptoms are the most important to address	A3.1 This requires integration of a number of different variables; motor/non motor symptoms, life habits of the PwP, adverse effects and the progression of the disease and then integrating that with the need to address symptoms deemed to be most salient/problematic by the PwP/Caregiver.	A3.1 This requires integration of a number of different variables; motor/non motor symptoms, life habits of the PwP, adverse effects and the progression of the disease and then integrating that with the need to address symptoms deemed to be most salient/problematic by the PwP/Caregiver.
B1 Establish adherence to existing Care Plan.	Is there evidence of non-adherence to prescribed pharmacological or supporting therapy care plan.	B1.1 Identifying non-adherence can be difficult to achieve via self-report. B1.2 Establishing whether symptoms are related to advances in disease state or non-adherence to prescribed Care Plan is important for future clinical decisions. B1.3 Identifying why non-adherence is occurring (i.e. barriers) in order to overcome them. This can be time consuming and is dependent on the social, educational, or cognitive status of the PwP/Caregiver.	B1.1 Identifying non-adherence can be difficult to achieve via self-report. B1.2 Establishing whether symptoms are related to advances in disease state or non-adherence to prescribed Care Plan is important for future clinical decisions. B1.3 Identifying why non-adherence is occurring (i.e. barriers) in order to overcome them. This can be time consuming and is dependent on the social, educational, or cognitive status of the PwP/Caregiver.
B2 Assess effectiveness of current medication/Adverse effects.	Is the medication they are currently taking providing the optimal Quality of Life for the PwP and how to balance the disease symptoms with drug induced adverse effects.	B2.1 Depending on the disease stage the PwP has reached and how long they have been on medication, the available effective pharmacological treatments become limited. B2.2 Making step-by-step adjustments to the medication in order to achieve an optimal solution is time consuming. B2.3 Lack of objective data to monitor the effects of changes made to the medication plan.	N/A
B3 Assess effectiveness of supporting therapies.	Are the Supporting therapies they are currently receiving adding value to the PwP’s overall QOL.	B3.1 Supporting therapies are not always an available option and if available can take time to access. B3.2 Even if a PwP has received a care plan from a supporting clinician it is difficult to establish their level of motivation/ adherence to that plan ongoing when at home.	B3.1 Supporting therapies are not always an available option and if available can take time to access. B3.2 Even if a PwP has received a care plan from a supporting clinician it is difficult to establish their level of motivation/ adherence to that plan ongoing when at home.
C1 Establish/Re-establish PwP goals in relation to Care Plan ongoing	Does the existing Care Plan allow the PwP to achieve their daily lifestyle goals.	C1.1 This requires integration of a number of different variables; motor/non motor symptoms, life habits of the PwP, adverse effects and the progression of the disease.	C1.1 This requires integration of a number of different variables; motor/non motor symptoms, life habits of the PwP, adverse effects and the progression of the disease.
C2 Identify barriers to existing care Plan/future Care Plan implementation by PwP	What are the likely barriers to adherence to existing/future Care Plan and how these can be overcome.	C2.1 This is dependent on the availability of data (via self-report) of previous adherence/non-adherence and is impacted by social, educational, or cognitive status of the PwP/Caregiver.	C2.1This is dependent on the availability of data (via self-report) of previous adherence/non-adherence and is impacted by social, educational, or cognitive status of the PwP/Caregiver.
D1 Prescribe pharmacological therapy(ies)	What are the optimal suite of drugs including appropriate dosage and format to prescribe ongoing.	D1.1 This requires integration of a number of different variables; motor/non motor symptoms, life habits of the PwP, adverse effects and the progression of the disease. D1.2 Depending on the disease stage the PwP has reached and how long they have been on medication, the available effective pharmacological treatments become limited. D1.3 This needs to be achieved by small ‘step by step’ changes personalized to a particular PwP. D1.4 Lack of objective data to monitor the effects of changes in the Care Plan.	N/A
D2 Prescribe Supporting Therapy(ies)	What Supporting Therapies ongoing would complement the prescribed drug therapy and add value to the PwP’s overall QOL.	D2.1 Supporting therapies are not always an available option and if available can take time to access. D2.2 Even if a PwP has received a care plan from a supporting clinician it is difficult to establish their level of motivation/ adherence to that plan ongoing when at home.	D2.1 Supporting therapies are not always an available option and if available can take time to access. D2.2 Even if a PwP has received a care plan from a supporting clinician it is difficult to establish their level of motivation/ adherence to that plan ongoing when at home.
E1 Establish timing for next consultation	What is the optimal time lapse before the next clinical consultation.	E1.1 Following up on ‘step by step’ changes to care plans can be difficult as typically clinician cannot see the PwP as frequently as they would like to in non-private settings. E1.2 This is dependent on the disease stage and whether the PwP is fluctuating/ unstable or in a relatively stable state but it can be difficult to establish this from self-report alone or from clinical examination during a given consultation.	E1.1 Following up on ‘step by step’ changes to care plans can be difficult as typically clinician cannot see the PwP as frequently as they would like to in non-private settings. E1.2 This is dependent on the disease stage and whether the PwP is fluctuating/ unstable or in a relatively stable state but it can be difficult to establish this from self-report alone or from clinical examination during a given consultation.
E2 Initiate referrals for Supporting Therapies defined in Care Plan	Is there sufficient justification/access to Supporting Therapies and how should this be achieved.	See D2.1 and D2.2	N/A
E3 Provide update/report to lead clinician and other MDT members	Identifying which pieces of clinical information need to be shared with the various team members in the MDT.	E3.1The sharing of clinical data between clinicians and across MDTs is typically achieved by a variety of methods such as telephone, clinical report, email and/or regular team meetings and is dependent on the context in which the care is being delivered.	E3.1 The sharing of clinical data between clinicians and across MDTs is typically achieved by a variety of methods such as telephone, clinical report, email and/or regular team meetings and is dependent on the context in which the care is being delivered.

## Appendix 2

**Table 6:** Perceived usefulness of objective data by Prescribing and Supporting Clinicians

	Prescribing Clinicians	Supporting Clinicians
Motor Symptoms		
Tremor	Important symptom in diagnosis and early stages of the disease. Can influence PwP QoL in some cases (although as disease progresses this is considered to be less useful than other motor symptoms data).	Important symptom for establishment of appropriate occupational therapy care plan Important symptom for establishment of appropriate physiotherapy care plan Can impact on psychological wellbeing (e.g. shame/ embarrassment)
Bradykinesia	Significantly influences PwP QoL. Directly impacts on the pharmacological decisions made. Is an indicator of the disease progression.	Important symptom for establishment of appropriate occupational therapy care plan Important symptom for establishment of appropriate physiotherapy care plan
Gait and Postural balance	Significantly influences PwP QoL. Directly impacts on the pharmacological decisions made. Important for the safety of the PwP (i.e. potential for increased falls/injury).	Important symptom for establishment of appropriate occupational care plan Important symptom for establishment of appropriate physiotherapy care plan Important for the safety of the PwP (i.e. potential for increased falls/injury).
On/Off fluctuations/ Freezing	Significantly influences PwP QoL. Directly impacts on the pharmacological decisions made. Is an indicator of the disease progression. Important for the safety of the PwP (i.e. increased falls/injury).	Important symptom for establishment of appropriate occupational therapy care plan Important symptom for establishment of appropriate physiotherapy care plan Can impact on psychological wellbeing (e.g. perception of shame/embarrassment) Relevant symptom for establishment of appropriate speech therapy care plan.
Dyskinesia	Significantly influences PwP QOL. Directly impact on the pharmacological decisions made. Is an indicator of the disease progression.	Important symptom for establishment of appropriate physiotherapy care plan Can impact on psychological wellbeing (e.g. shame/ embarrassment)
Speech disorders	Important symptom in diagnosis and early stages of the disease Can influence PwP QOL in some cases (although as disease progresses this is considered to be less useful than other motor symptoms data).	Important symptom for establishment of appropriate speech therapy care plan Can impact on psychological wellbeing (e.g. shame/ embarrassment)
Muscle Rigidity	Significantly influences PwP QoL. Directly impacts on the pharmacological decisions made.	*Not specifically commented on by Supporting Clinicians*
Dysphagia	Significantly influences PwP QoL.	Significantly influences PwP QoL. Relevant symptom for establishment of appropriate speech therapy care plan
Non-motor symptoms		
Cognitive Disorders/Dementia	Significantly influences PwP QoL. Directly impacts on the pharmacological decisions made. Is an indicator of the disease progression. Can impact on psychological wellbeing (e.g. shame/ embarrassment)	Important symptom for establishment of appropriate occupational therapy care plan. Important symptom for establishment of appropriate physiotherapy care plan Important symptom for establishment of appropriate psychological care plan
Depression/Anxiety	Significantly influences PwP QoL. Directly impacts on the pharmacological decisions made. Is an indicator of the disease progression.	Important symptom for establishment of appropriate occupational therapy care plan Important symptom for establishment of appropriate physiotherapy care plan Important symptom for establishment of appropriate psychological care plan Relevant symptom for establishment of appropriate speech therapy care plan
Impulse Control disorders	Significantly influences PwP QoL. Directly impacts on the pharmacological decisions made.	Important symptom for establishment of appropriate occupational therapy care plan Important symptom for establishment of appropriate physiotherapy care plan Important symptom for establishment of appropriate psychological care plan Relevant symptom for establishment of appropriate speech therapy care plan.
Sleep Disorders/ Daytime Sleepiness	Significantly influences PwP QoL. Can impact on the pharmacological decisions made.	Important symptom for establishment of appropriate occupational therapy care plan Important symptom for establishment of appropriate physiotherapy care plan Important symptom for establishment of appropriate psychological care plan
Dizziness/Fainting	Significantly influences PwP QOL. Can impact on the pharmacological decisions made. Important for the safety of the PwP (i.e. increased falls/injury).	Important symptom for establishment of appropriate occupational care plan Important symptom for establishment of appropriate physiotherapy care plan Important for the safety of the PwP (i.e. potential for increased falls/injury).
Fatigue	Significantly influences PwP QoL. Can impact on the pharmacological decisions made.	Important symptom for establishment of appropriate occupational therapy care plan Important symptom for establishment of appropriate physiotherapy care plan Important symptom for establishment of appropriate psychological care plan
Sweating	Can influence QoL for a minority of PwPs	Can influence QoL for a minority of PwPs
Urinary/ Constipation Problems	Significantly influences PwP QoL.	Important symptom for establishment of appropriate physiotherapy care plan Important symptom for establishment of appropriate nutritional care plan
Nausea/ Gastro-intestinal problems	Can influence QoL for some PwPs	Important symptom for establishment of appropriate nutritional care plan
Pain	Can influence QoL for a minority of PwPs	Can influence QoL for a minority of PwPs
Hallucination incidence	Can influence QoL for a minority of PwPs	Can influence QoL for a minority of PwPs

## Appendix 3

**Table 7:** Prescribing Clinicians’ User Interfaces – Proposed data requirements per goal related domains

	Suggested data provision for each User Interface Domain				
	UI_1A Diagnosis Indicators	UI_1B Disease Progression Indicators	UI_1C Pharmacological Decision Support	UI_1D PwP Safety	UI_1E Overall QoL
Motor Symptoms Data					
Tremor	**√**				**√**
Muscle Rigidity	**√**	**√**	**√**		
Bradykinesia	**√**	**√**	**√**		
Gait/Postural Balance	**√**	**√**	**√**	**√**	
Dyskinesia		**√**	**√**		
On Off fluctuations/ Freezing		**√**	**√**	**√**	**√**
Speech Disorder	**√**				**√**
Dysphagia		**√**	**√**		**√**
Non-motor Symptoms Data					
Cognitive Disorders/Dementia	**√**	**√**	**√**		**√**
Depression and Anxiety		**√**	**√**		**√**
Impulse control disorders			**√**		**√**
Sleep disorders/Daytime sleepiness			**√**		**√**
Dizziness/Fainting			**√**	**√**	
Fatigue					**√**
Sweating					**√**
Urinary/ Constipation Problems					**√**
Nausea/ Gastro-intestinal problems					**√**
Other Supporting Data					
Video of PwP symptoms	**√**	**√**	**√**		**√**
Speech quality recordings		**√**			**√**
Overall activity levels		**√**	**√**	**√**	**√**
Number of falls/time		**√**	**√**	**√**	**√**
Pain index			**√**		**√**
Hallucination incidence		**√**	**√**		**√**
Blood Pressure/time		**√**	**√**	**√**	
Bodyweight/time					**√**
Daily fluid intake					**√**
Adherence to care plans					
Pharmacological		**√**	**√**		
Nutritional		**√**	**√**		
Physiotherapy		**√**	**√**		
Speech		**√**	**√**		

**Table 8:** Supporting Clinicians’ User Interfaces – Proposed data requirements per discipline

	Suggested data provision for each User Interface Domain		
	UI_2A Physiotherapy and Occupational Therapy	UI_2B Psychology	UI_2C Speech therapy
Motor Symptoms Data			
Tremor	**√**	**√**	
Muscle Rigidity	**√**		
Bradykinesia	**√**		
Gait/Postural Balance	**√**		
Dyskinesia	**√**	**√**	
On Off fluctuations/ Freezing	**√**	**√**	**√**
Speech Disorder	**√**	**√**	**√**
Dysphagia	**√**	**√**	**√**
Non-motor Symptoms Data			
Cognitive Disorders/Dementia	**√**	**√**	**√**
Depression and Anxiety	**√**	**√**	**√**
Impulse control disorders	**√**	**√**	
Sleep disorders/Daytime sleepiness	**√**	**√**	
Dizziness/Fainting	**√**		
Fatigue	**√**	**√**	
Sweating			
Urinary/ Constipation Problems	**√**		
Nausea/ Gastro-intestinal problems			
Other Supporting Data			
Video of PwP symptoms	**√**		**√**
Speech quality recordings			**√**
Overall activity levels	**√**		
Number of falls/time	**√**		
Pain index	**√**		
Hallucination incidence		**√**	
Blood Pressure/time			
Bodyweight/time	**√**		
Daily fluid intake			
Adherence to care plans			
Pharmacological	**√**	**√**	**√**
Nutritional	**√**	**√**	**√**
Physiotherapy	**√**	**√**	**√**
Speech	**√**	**√**	**√**

## Appendix 4

**Table 9:** User derived requirements for PD_manager design

Requirement	Details
R1	Motor and non-motor symptom outputs should be in a quick and easy to understand graphical format.
R2	Clinician needs to be able to easily identify/compare changes in symptoms overtime and drill down into different time periods depending on the needs of the patient.
R3	Provide video capture data to help establish the difference between Dyskinesia and Tremor in patients’ self-report.
R4	Data provision should facilitate the ability to establish the co-occurrence of, and comparison of, motor with non-motor symptoms
R5	The User Interface should provide flexibility for the clinician to choose, in conjunction with the patient which symptoms and data collection options/time periods are of most interest for a particular patient and be able to explore these in the context of a personalised integrated data output.
R6	Provide the clinician and patient/caregiver with data on patient’s adherence to pharmacological and supporting therapy care plans over time.
R7	Provide the clinician and patient/caregiver access to an up-to-date list of a patient’s current prescribed pharmacological care plan (e.g. drug name, format, dosage etc.).
R8	Provide the clinician and patient/caregiver with access to an up-to-date list of patient’s current prescribed supporting therapy care plans so that they have a view of all the activities that should be taking place i.e. for a GP it would be useful to be able to see whether a PD nurse specialist had prescribed changes to a care plan. Similarly it would be useful for a physiotherapist to be able to see what occupational therapy plans had been prescribed etc. thus facilitating a better understanding of the overall care being provided across the multidisciplinary team (MDT).
R9	Provide the clinician with the ability to monitor the effectiveness of the step-by-step changes made to pharmacological care plans both at and between face-to-face consultations (i.e. remotely) to establish if the change has resulted in a positive/negative outcome.
R10	Provide the clinician and patient/caregivers with the ability to access data on patient’s activity levels/duration in their home environment.
R11	Provide the clinician with the ability to prescribe supporting therapies a patient can engage with at home and provide data on patient’s adherence/performance indicators when they have engaged with these therapies via the PD_manager platform (e.g. gamification of physio, cognitive activities, speech and nutrition).
R12	Provide a communication platform to facilitate the sharing of information and alerts between clinicians participating in the care of a patient in the context of an MDT.
R13	Provide functionality to alert patient/caregiver with when they should take their medication.

## Abbreviations

CDSS: Clinical Decision Support System
HTA: Hierarchical Task Analysis
HTD: Hierarchical Task Diagram
mHealth: Mobile Health
MRI: Magnetic Resonance Imagery
PD: Parkinson’s Disease
QoL: Quality of Life
QoS: Quality of Service
